# Design and characterization of protein-quercetin bioactive nanoparticles

**DOI:** 10.1186/1477-3155-9-19

**Published:** 2011-05-17

**Authors:** Ru Fang, Hao Jing, Zhi Chai, Guanghua Zhao, Serge Stoll, Fazheng Ren, Fei Liu, Xiaojing Leng

**Affiliations:** 1 CAU and ACC Joint Laboratory of Space Food, College of Food Science and Nutritional Engineering, China Agricultural University, Key Laboratory of Functional Dairy Science of Beijing and the Ministry of Education, Beijing Higher Institution Engineering Research Center of Animal Product, No.17 Qinghua East Road, Haidian, Beijing 100083, China; 2 Groupe de Physico-Chimie de L'Environnement, Institut Forel, Section des Sciences de la Terre et de l'Environnement, Université de Genève, 10, route de Suisse, CH-1290 Versoix, Switzerland

## Abstract

**Background:**

The synthesis of bioactive nanoparticles with precise molecular level control is a major challenge in bionanotechnology. Understanding the nature of the interactions between the active components and transport biomaterials is thus essential for the rational formulation of bio-nanocarriers. The current study presents a single molecule of bovine serum albumin (BSA), lysozyme (Lys), or myoglobin (Mb) used to load hydrophobic drugs such as quercetin (Q) and other flavonoids.

**Results:**

Induced by dimethyl sulfoxide (DMSO), BSA, Lys, and Mb formed spherical nanocarriers with sizes less than 70 nm. After loading Q, the size was further reduced by 30%. The adsorption of Q on protein is mainly hydrophobic, and is related to the synergy of Trp residues with the molecular environment of the proteins. Seven Q molecules could be entrapped by one Lys molecule, 9 by one Mb, and 11 by one BSA. The controlled releasing measurements indicate that these bioactive nanoparticles have long-term antioxidant protection effects on the activity of Q in both acidic and neutral conditions. The antioxidant activity evaluation indicates that the activity of Q is not hindered by the formation of protein nanoparticles. Other flavonoids, such as kaempferol and rutin, were also investigated.

**Conclusions:**

BSA exhibits the most remarkable abilities of loading, controlled release, and antioxidant protection of active drugs, indicating that such type of bionanoparticles is very promising in the field of bionanotechnology.

## Background

Over the last several decades, the development of nanoparticles as drug delivery systems has gained considerable interest. Nanotoxicology research has indicated that [1] not only pharmacological properties but also the biodegradability, biocompatibility, and nontoxicity should be considered in such new systems. Therefore, synthetic macromolecules, such as the amphiphilic hyperbranched multiarm copolymers (HPHEEP-star-PPEPs) [2], poly(2-ethyl-2-oxazoline)-b-poly(D,L-lactide) [3], and polyethylene glycol [4], are often investigated; replacing these synthetic materials with natural proteins, which are more likely to be accepted by people, has become the focus of many research studies [5-9]. However, the microstructure of natural substances is generally complex and difficult to control; progress largely depends on knowledge of the physiochemical properties of the materials.

The potential therapeutic usefulness of albumin, such as bovine serum albumin (BSA), is high; it possesses the ability to transport fatty acids and many other endogenous or exogenous compounds throughout the body [10,11]. Using a coacervation process, i.e., desolvation with ethanol and then solidification with glutaraldehyde, BSA can form nanoparticles [7]. Hydrophilic drugs, such as phosphodiester oligonucleotide, 5-fluorouracil, and sodium ferulate, among others, can be incorporated into the matrix or adsorbed on the surface of nanoparticles [7-9]. However, the molecular sizes obtained from such a process are often larger than 70 nm; such particles cannot be used to entrap hydrophobic drugs, thereby restricting the development of bio-nanocarriers.

**Figure 1:**
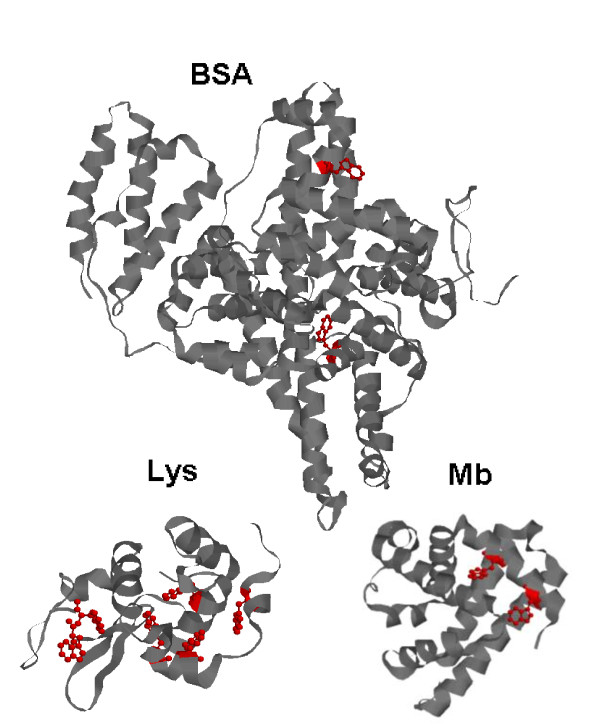
**Schematic drawing of the Lys, Mb, and BSA molecules**. Trp residues are marked in red.

The present study proposes a novel method for designing a small bioactive nanoparticle using BSA as a carrier to deliver hydrophobic drugs. Quercetin (Q), a polyphenol widely distributed in vegetables and plants, is used here as a model of hydrophobic drugs. Q exhibits anti-oxidative, free radical scavenging, anticancer, and antiviral activities [12]. However, the poor solubility and low stability of Q in aqueous alkaline medium [13] restrict the application of this type of drug in oral use. Dimethyl sulfoxide (DMSO), one of the most versatile organic solvents in biological science that can accept hydrogen-bond and interact with the hydrophobic residues of proteins [14], is used here to dissolve Q, and synthesize a novel nanocarrier with interesting drug delivery capabilities. Some studies have reported that BSA interacts with Q through tryptophan (Trp) [15,16]. BSA is a monomeric globular protein formed from 583 amino acid residues, containing two Trps, one of which is located in the inner hydrophobic pocket, corresponding to the so-called site II. Site II is a specific site for hydrophobic drugs due to its hydrophobicity [11,17]. To confirm the feasibility of the Trp transport functionality, lysozyme (Lys) and myoglobin (Mb) were also used in this work for comparison with BSA. Figure 1 exhibits the molecular structures of Lys, Mb, and BSA. Lys is a small monomeric globular protein formed from 129 amino acid residues, and contains six Trps. This protein is known to bind various small ligands, such as metal ions, non-metal ions, dyes, and numerous pharmaceuticals [18-20]. Mb is a small heme protein for oxygen storage and transport. It contains a single polypeptide chain of 153 amino acid residues and two Trps. The polypeptide chain provides a nonpolar pocket to accommodate and stabilize the porphyrin ring [21-23].

In the present study, the Q binding and releasing capacity of Lys and Mb are compared with those of BSA. The salting out method was combined with UV-Vis spectrometry to determine the binding capacity of the proteins. The release of Q from nanocarriers was detected in acidic and neutral conditions. The antioxidant properties of the bound Q in proteins were evaluated by 2,2-diphenyl-1-picrylhydrazyl (DPPH) and 2,2'-azino-bis(3-ethylbenzothiazoline-6-sulfonic acid) (ABTS) radicals. Raman, fluorescence, and UV-Vis spectroscopy were combined to study the secondary and tertiary structures of the protein aggregates.

## Results and Discussion

### Size and Zeta Potential Measurements

**Figure 2:**
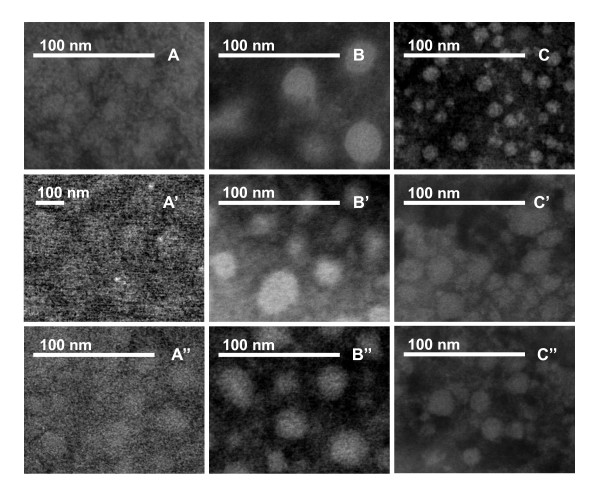
**STEM images of BSA, Lys, and Mb system**. The concentration of BSA, Lys, or Mb was 1.5 × 10^-5 ^mol/L. (A) Native BSA, no DMSO and Q were added; (B) 10% DMSO and BSA; (C) 10% DMSO, 1.5 × 10^-4 ^mol/L Q and BSA; (A') Native Lys, no DMSO and Q were added; (B') 10% DMSO and Lys; (C') 10% DMSO, 1.5 × 10^-4 ^mol/L Q and Lys; (A'') Native Mb, no DMSO and Q were added; (B'') 10% DMSO and Mb; (C'') 10% DMSO, 1.5 × 10^-4 ^mol/L Q and Mb.

**Figure 3:**
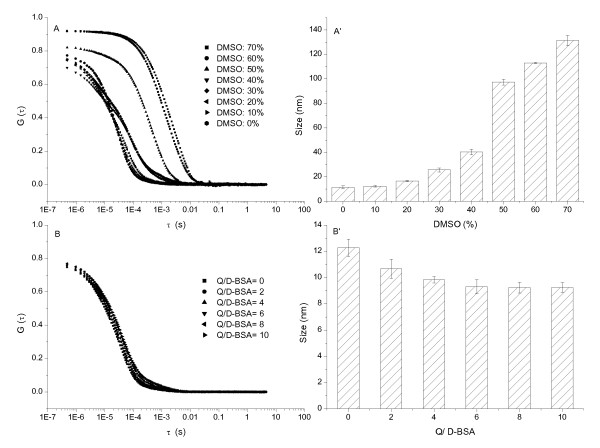
**DLS measurements of the BSA system**. The concentration of BSA was 1.5 × 10^-5 ^mol/L. (A) ACF of BSA vs. the concentration of DMSO; (A') Size distribution histogram of BSA vs. the concentration of DMSO; (B) ACF of BSA vs. the concentration of Q; (B') Size distribution histogram of BSA vs. the concentration of Q. The concentration of DMSO was maintained at 10% in B and B'.

**Figure 4:**
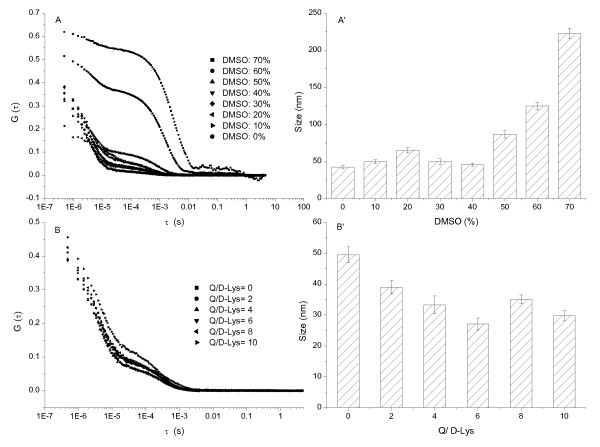
**DLS measurements of the Lys system**. The concentration of Lys was 1.5 × 10^-5 ^mol/L. (A) ACF of Lys vs. the concentration of DMSO; (A') Size distribution histogram of Lys vs. the concentration of DMSO; (B) ACF of Lys vs. the concentration of Q; (B') Size distribution histogram of Lys vs. the concentration of Q. The concentration of DMSO was maintained at 10% in B and B'.

Scanning transmission electron microscopy (STEM) and dynamic light scattering (DLS) were combined to analyze the size and conformational features of the BSA, Lys, and Mb systems, as shown in Figures 2, 3, 4, &5. STEM micrographs show that the native BSA, Lys, and Mb molecules (without DMSO) were cross-linked, and formed loose aggregates (Figures 2A, **A'**, and **A''**). When the added amount of DMSO was over 10% (v/v), DMSO-inducing protein (BSA, Lys, or Mb) nanoparticles (D-BSA, D-Lys, or D-Mb) formed, showing compact and spherical aggregates (Figures 2B, B', and 2B''). After adding 1.5 × 10^-4 ^mol/L Q solution prepared with 10% DMSO, spherical and compact Q loaded protein (BSA, Lys, or Mb) nanoparticles (D-BSA-Q, D-Lys-Q, or D-Mb-Q) also occurred (Figures 2C, C', and 2C''), but their size decreased compared with the system without Q, particularly the D-BSA-Q aggregates, which markedly decreased in size.

**Figure 5:**
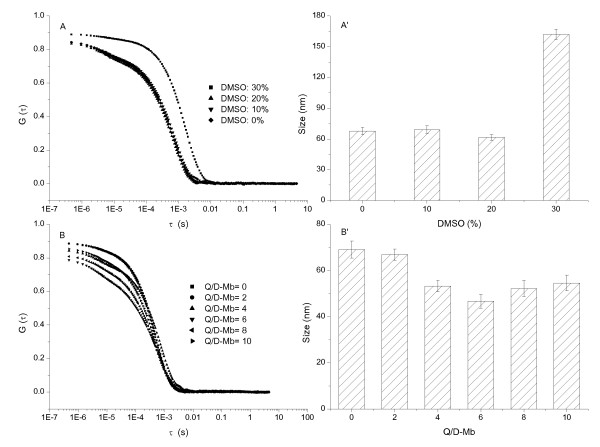
**DLS measurements of the Mb system**. The concentration of Mb was 1.5 × 10^-5 ^mol/L. (A) ACF of Mb vs. the concentration of DMSO; (A') Size distribution histogram of Mb vs. the concentration of DMSO; (B) ACF of Mb vs. the concentration of Q; (B') Size distribution histogram of Mb vs. the concentration of Q. The concentration of DMSO was maintained at 10% in B and B'.

The autocorrelation function curve (ACF) of light scattering, G(τ) (τ is delay time), was used to determine the hydrodynamic particle sizes of the system [24,25]. The size of D-BSA (Figures 3A and 3A') and D-Lys (Figures 4A and 4A') was less than 50 nm when the concentration of DMSO was less than 40%; this increased markedly with increasing DMSO concentrations. The size of D-Mb was maintained at about 70 nm when the DMSO concentration was less than 20%; serious precipitation is produced with concentrations of DMSO over 40% (Figures 5A and 5A'). Therefore, the concentration of DMSO was maintained at 10%, but the concentration of Q was changed. The sizes of D-BSA-Q (Figures 3B and 3B'), D-Lys-Q (Figures 4B and 4B'), and D-Mb-Q (Figures 5B and 5B') became smaller than those of D-BSA, D-Lys, and D-Mb, respectively. Moreover, the sizes of both D-Lys-Q and D-Mb-Q were generally larger than D-BSA-Q. These observations were in accordance with the STEM analysis.

**Figure 6:**
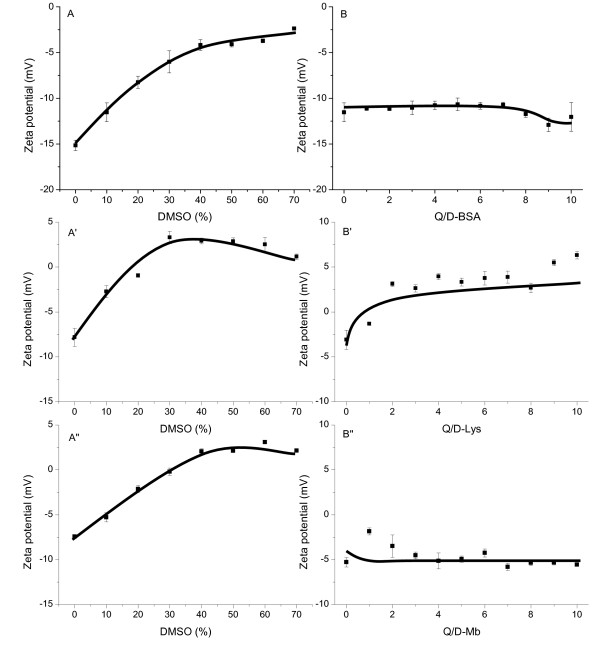
**Zeta potential measurements of BSA, Lys, and Mb systems**. The concentration of BSA, Lys, or Mb was 1.5 × 10^-5 ^mol/L. (A) Zeta potential of BSA vs. the concentration of DMSO; (B) Zeta potential of BSA vs. the concentration of Q. (A') Zeta potential of Lys vs. the concentration of DMSO; (B') Zeta potential of Lys vs. the concentration of Q. (A'') Zeta potential of Mb vs. the concentration of DMSO; (B'') Zeta potential of Mb vs. the concentration of Q. The concentration of DMSO was kept constant at 10% in B, B', and B''. Solid lines were used to illustrate the trends of the experimental data (in symbols) in both A, A', A'',B, B', and B''.

Figure 6 shows the variation of the zeta potential of the BSA, Lys, and Mb systems versus the concentration of DMSO (**A, A'**, and **A''**) and Q (**B, B'**, and **B''**). With increasing DMSO concentration, the zeta potential values of D-BSA, D-Lys, and D-Mb tended to decline to zero (**A, A' **and **A''**). The loss of surface charges indicates that the protein aggregations were caused by the gradually enhanced hydrophobic forces compared with electrostatic ones. Upon addition of Q, the zeta potential values of D-BSA-Q, D-Lys-Q, and D-Mb-Q became -12.5, 2.5, and -5 mV (**B, B'**, and **B''**), respectively. Size analysis showed that D-BSA-Q, D-Lys-Q, and D-Mb-Q were smaller than D-BSA, D-Lys, and D-Mb, respectively, indicating that protein aggregation was hindered by electrostatic repulsion in these systems compared with the system without Q. The corresponding potential variations could be related to the features of the amino acid residues of the polypeptide backbone and protein structural transformation caused by Q. To attain a better understanding of the changes in the secondary and tertiary structures of the protein molecules during aggregation, Raman, fluorescence, and UV-Vis spectroscopy were performed. The molecular mass of native BSA, Lys, and Mb molecules (M_BSA_, M_Lys_, and M_Mb_), D-BSA-Q, D-Lys-Q, and D-Mb-Q prepared with 1.5 × 10^-4 ^mol/L Q and 10% DMSO (M_D-BSA-Q_, M_D-Lys-Q_, and M_D-Mb-Q_), were determined using the DLS method. The ratio of M_D-BSA-Q _/ M_BSA _obtained was found to vary between 1.1 and 2.2, indicating that one BSA nanocarrier consisted of not more than 2 BSA molecules. However, the obtained ratios of M_D-Lys-Q _/ M_Lys _and M_D-Mb-Q _/ M_Mb _were 4.8 and 5.1, respectively, indicating that one Lys nanocarrier consisted of more than 4 Lys molecules, and one Mb nanocarrier consisted of more than 5 Mb molecules.

### Laser Raman spectroscopy

**Figure 7:**
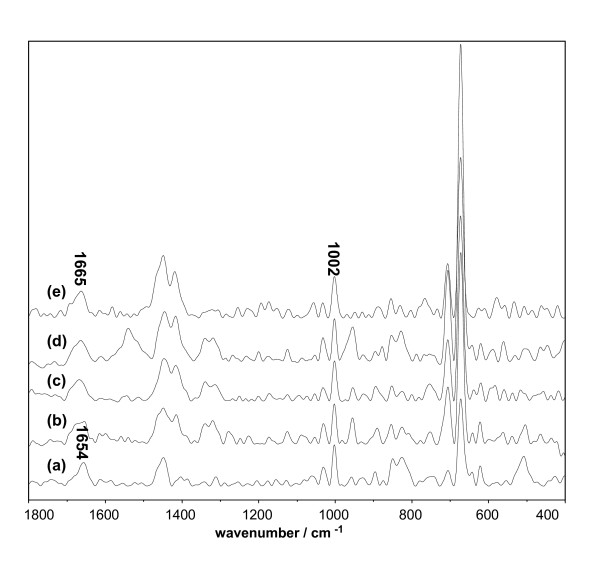
**Raman spectrum of BSA system vs. the concentration of DMSO**. The concentration of BSA was 1.5 × 10^-5 ^mol/L. (a) Native BSA; (b) BSA and 10% DMSO; (c) BSA and 30% DMSO; (d) BSA and 50% DMSO; (e) BSA and 70% DMSO.

**Figure 8:**
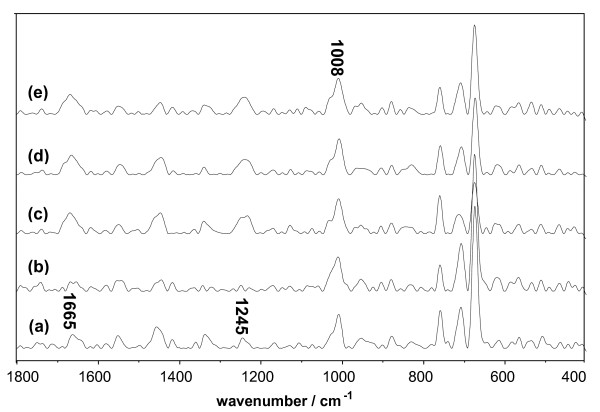
**Raman spectrum of Lys system vs. the concentration of DMSO**. The concentration of Lys was 1.5 × 10^-5 ^mol/L. (a) Native Lys; (b) Lys and 10% DMSO; (c) Lys and 30% DMSO; (d) Lys and 50% DMSO; (e) Lys and 70% DMSO.

**Figure 9:**
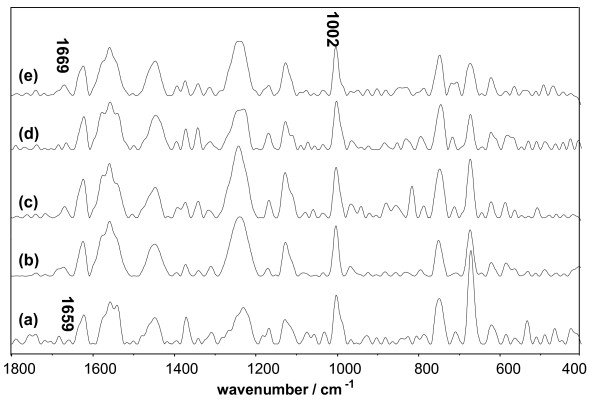
**Raman spectrum of Mb system vs. the concentration of DMSO**. The concentration of Mb was 1.5 × 10^-5 ^mol/L. (a) Native Mb; (b) Mb and 10% DMSO; (c) Mb and 30% DMSO; (d) Mb and 50% DMSO; (e) Mb and 70% DMSO.

Raman spectroscopy was employed to investigate changes in the secondary and tertiary structures of the protein molecules during aggregation. Figure 7 compares the Raman spectra of native BSA and D-BSA in the 1800-400 cm^-1 ^region. Consistent with the literature [26,27], the secondary structure of native BSA was largely α-helical in form; this was supported by an amide I signal at 1654 cm^-1^. The decrease in band intensity with DMSO concentration presented in Table 1 indicates the loss of the α-helix during aggregation. Meanwhile, the broadening of this band and the increase of the band intensity at 1665 cm^-1 ^implies the increase of the random-coil content in the protein structure [26].The coincident trends were observed in Lys (Figure 8) and Mb (Figure 9) systems. Over 30% of the secondary structure of native Lys presented in random coil conformation, as supported by an amide I signal at 1665 cm^-1 ^and an amide III signal at 1245 cm^-1^. The change in intensity of these bands, presented in Table 2, shows the increase of random-coil in protein microstructures with DMSO. The secondary structure of the native Mb was largely α-helical in form, as supported by an amide I signal at 1659 cm^-1^. Similar to the case of D-BSA, the disappearance of this band with DMSO concentration, presented in Table 3, indicates the decrease of α-helix during aggregation. The increase in intensity of the band at 1669 cm^-1 ^implies an increase in random-coil content in the protein structure during aggregation. The loss of the α-helix is attributed to the competition between the S = O group of DMSO and the C = O groups of protein for the amide's hydrogen molecules, resulting in the partial unfolding of the polypeptide chain, exposure of the internal hydrophobic groups, and promotion of protein aggregation by hydrophobic effects and H-bonding [14,28]. This belief is supported by the zeta potential measurements in the previous section.

**Table 1:** Intensities^a ^of Raman Band of BSA system

	1665 cm^-1^	1654 cm^-1^
BSA	N. D.	0.54
BSA + DMSO (10%)	0.31	0.34
BSA + DMSO (30%)	0.36	0.23
BSA + DMSO (50%)	0.39	0.22
BSA + DMSO (70%)	0.41	N. D.

^a^Integrated intensity (peak intensity) relative to that of the phenylalanine band at 1002 cm^-1^. N. D. = not detected. The concentration of BSA was 1.5 × 10^-5 ^mol/L.

**Table 2:** Intensities^a ^of Raman Band of Lys system

	1665 cm^-1^	1245 cm^-1^
Lys	0.41	0.31
Lys + DMSO (10%)	0.27	0.17
Lys + DMSO (30%)	0.60	0.47
Lys + DMSO (50%)	0.55	0.42
Lys + DMSO (70%)	0.56	0.48

^a^Integrated intensity (peak intensity) relative to that of the phenylalanine band at 1008 cm^-1^. The concentration of Lys was 1.5 × 10^-5 ^mol/L.

**Table 3:** Intensities^a ^of Raman Band of Mb system

	1669 cm^-1^	1659 cm^-1^
Mb	N.D.	0.08
Mb + DMSO (10%)	0.18	N.D.
Mb + DMSO (30%)	0.22	N.D.
Mb + DMSO (50%)	0.14	N.D.
Mb + DMSO (70%)	0.22	N.D.

^a^Integrated intensity (peak intensity) relative to that of the phenylalanine band at 1002 cm^-1^. N. D. = not detected. The concentration of Mb was 1.5 × 10^-5 ^mol/L.

**Figure 10:**
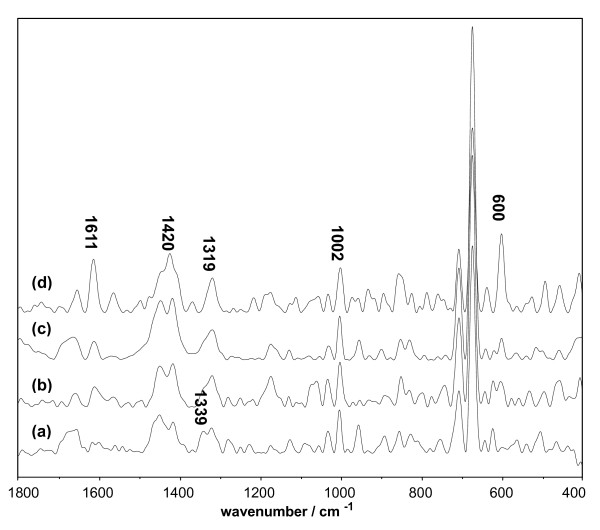
**Raman spectrum of BSA system vs. the concentration of Q**. The concentrations of BSA and DMSO were maintained at 1.5 × 10^-5 ^mol/L and 10%, respectively. (a) 0 mol/L Q; (b) 3.0 × 10^-5 ^mol/L Q; (c) 9.0 × 10^-5 ^mol/L Q; (d) 1.5 × 10^-4 ^mol/L Q.

**Figure 11:**
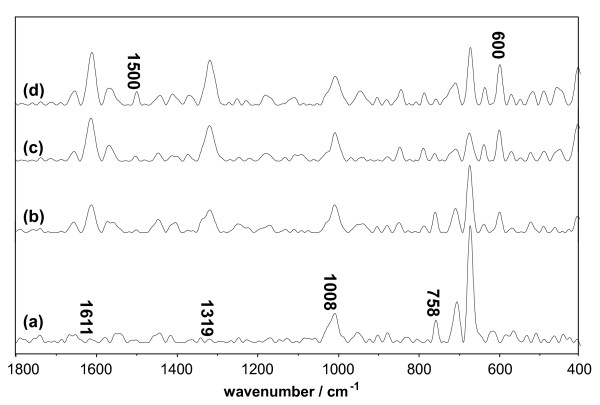
**Raman spectrum of Lys system vs. the concentration of Q**. The concentrations of Lys and DMSO were maintained at 1.5 × 10^-5 ^mol/L and 10%, respectively. (a) 0 mol/L Q; (b) 3.0 × 10^-5 ^mol/L Q; (c) 9.0 × 10^-5 ^mol/L Q; (d) 1.5 × 10^-4 ^mol/L Q.

The Raman spectra of D-BSA-Q and D-Lys-Q are shown in Figures 10 and 11, respectively; here, the concentration of DMSO was kept constant at 10%. The band at 1611 cm^-1 ^(Figures 10 and 11), which is sensitive to the bound ligands, is a marker of the orientation of the indole ring of Trp with respect to the Cα atom of the peptide backbone [29]. The increase in band intensities shown in Tables 4 and 5 indicates that the added Q led to the reorientation of the indole ring through the adjustment in the torsional angle of the side chain. The bands near 1319 and 600 cm^-1 ^were ascribed to aliphatic CH_2 _twisting deformations and the pyrrole ring skeletal of Trp [30], respectively. The significant increase in their intensities with increasing Q proved the interactions between Trp and Q (Figures 10 and 11, Tables 4 and 5). The bands near 1339 [31,32] and 758 [33] cm^-1 ^have been found to be indicators of the hydrophobicity of the Trp environment, and a decrease in these band intensities (Figures 10 and 11, Tables 4 and 5) indicates that the molecular environment of Trp is more hydrophobic due to the interactions between the indole ring and Q.

**Table 4:** Intensities^a ^of Raman Band in BSA

	1613 cm^-1^	1420 cm^-1^	1339 cm^-1^	1319 cm^-1^	600 cm^-1^
D-BSA	0.20	1.01	0.51	0.59	0.12
D-BSA + Q2	0.49	1.13	0.46	0.73	0.54
D-BSA + Q6	0.42	1.40	N. D.	0.69	0.49
D-BSA + Q10	1.15	1.32	N. D.	0.78	1.72

^a^Integrated intensity (peak intensity) relative to that of the phenylalanine band at 1002 cm^-1^. N. D. = not detected. The concentration of BSA was 1.5 × 10^-5 ^mol/L, and DMSO was kept at 10%. Q2, Q6, and Q10 indicate concentrations of Q at 3.0 × 10^-5^, 9.0 × 10^-5^, and 15.0 × 10^-5 ^mol/L, respectively.

The intensity of the band near 1420 cm^-1^, which was observed in the Raman spectra of D-BSA-Q (Table 4), increased with Q, indicating exposure of the ionized carboxyl group (COO^-^) of aspartic (Asp) and glutamic acid (Glu) residues [29,34,35], the PK_α _values of which are 3.9 and 4.3, respectively. These resulted in the negative charges of the particles. The intensity of the band at 1500 cm^-1 ^increased with Q (Table 5), indicating exposure of the ionized amino group (NH_3_^+^) of lysine (Lys) and arginine (Arg) residues, the PK_α _values of which are 10.5 and 12.5, respectively [36]. These resulted in the positive charges of the particles. The negative or positive charges weakened the tendency of the particles to undergo aggregation. This conclusion is in agreement with the zeta potential measurements in the previous section.

**Table 5:** Intensities^a ^of Raman Band in Lys

	1611 cm^-1^	1500 cm^-1^	1319 cm^-1^	758 cm^-1^	600 cm^-1^
D-Lys	0.13	0.09	0.12	0.77	0.01
D-Lys+ Q2	1.00	0.10	0.82	0.74	0.74
D-Lys+ Q6	1.51	0.18	1.25	0.27	1.09
D-Lys+ Q10	1.83	0.47	1.56	0.22	1.40

^a^Integrated intensity (peak intensity) relative to that of the phenylalanine band at 1008 cm^-1^. The concentration of Lys was 1.5 × 10^-5 ^mol/L, and DMSO was kept at 10%. Q2, Q6, and Q10 indicate concentrations of Q at 3.0 × 10^-5^, 9.0 × 10^-5^, and 15.0 × 10^-5 ^mol/L, respectively.

**Figure 12:**
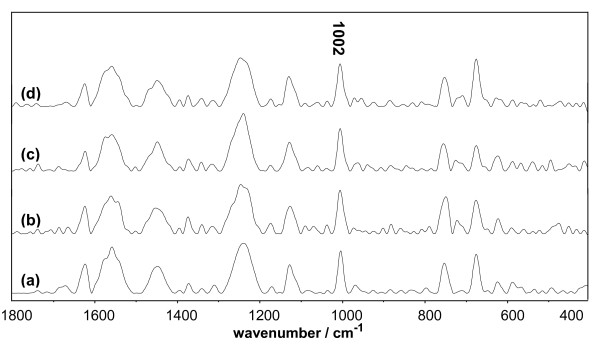
**Raman spectrum of Mb system vs. the concentration of Q**. The concentrations of Mb and DMSO were maintained at 1.5 × 10^-5 ^mol/L and 10%, respectively. (a) 0 mol/L Q; (b) 3.0 × 10^-5 ^mol/L Q; (c) 9.0 × 10^-5 ^mol/L Q; (d) 1.5 × 10^-4 ^mol/L Q.

Mb consists of eight helical regions and a non-covalent bound heme prosthetic group, which is buried in a relatively hydrophobic pocket interior of the protein. With laser excitation, the Raman bands of the porphyrin skeleton, appearing between 1650 and 1100 cm^-1^, become very intense and disturb the signals of the other bands (Figure 12). This phenomenon brings difficulty in the analysis in this region [21,37]. In addition, the approach of two Trp residues to the heme results in a partial energy transfer of the chromophoric group in Trp [37], and causes the Raman bands arising from Trp, such as those at 1611, 1319, and 600 cm^-1^, to become very weak (Figure 12).

### Fluorescence Spectroscopy

**Figure 13:**
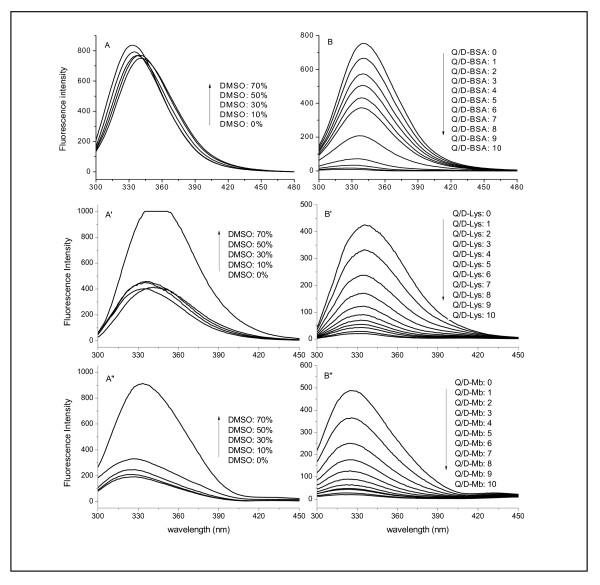
**Fluorescence emission spectra of BSA, Lys, and Mbsystem**. The concentration of (A and B) BSA, (A' and B') Lys, or (A'' and B'') Mb was 1.5 × 10^-5 ^mol/L. (A), (A'), and (A'') Effects of DMSO at 27°C. (B), (B'), and (B'') Effects of Q at 27°C. DMSO was maintained at 10%.

Figure 13 compares the fluorescence spectra of the D-BSA (**A**), D-Lys (**A'**), D-Mb (**A''**), D-BSA-Q (**B**), D-Lys-Q (**B'**), and D-Mb-Q (**B''**) versus the concentration of DMSO or Q. At an excitation wavelength of 280 nm, native BSA and Lys showed maximum intrinsic fluorescence at 340 nm, while Mb showed a maximum at 328 nm; these are believed to be caused by Trp residues. Of the two Trp residues in BSA, one is located near the surface of the protein molecule; in the case of Lys [38] and Mb [37], three and one Trp residues are respectively located near the surfaces of the molecules. The fluorescence of tyrosine (Tyr) residues (304 nm) was extremely weak and could be neglected. A slight increase in the intensity of fluorescence, as well as a blue shift, was observed when the concentration of DMSO in the BSA and Lys systems was less than 70% (Figures 13A and **A'**); this indicates that the microenvironment of Trp residues was more hydrophobic. In the case of Mb, a slight increase in fluorescence intensity also occurred, but a red shift, rather than a blue one, was observed (Figure 13A''). This suggests that the Trp residues in Mb were more hydrophilic. These phenomena may have resulted from structural changes in the proteins. When the concentration of DMSO was increased to 70%, a sharp increase in the fluorescence intensity in the Lys and Mb systems (Figures 13A' and **A''**) was observed, indicating that the surface Trp residues were buried into the protein aggregates [39-41].

With the addition of Q, fluorescence quenching was observed in D-BSA, D-Lys, and D-Mb; simultaneous slight blue shifts also occurred (Figures 13B, 13B', and 13B''). Quenching processes usually involve two modes, dynamic and static. Dynamic quenching occurs when the excited fluorophore experiences contact with an atom or molecule that can facilitate non-radiative transitions to the ground state, while static quenching implies either the existence of a spherical region of effective quenching, or the formation of a ground-state non-ﬂuorescent complex. In many cases, the fluorophore can be quenched both by collision and by complex formation with the same quencher [42,43]. The binding of Q with BSA, Lys, or Mb was static, as Q was less than 1.5 × 10^-5 ^mol/L. The mode was determined by comparing the fitting results of the dynamic, static, and the combination modes to the D-BSA-Q, D-Lys-Q, and D-Mb-Q systems (See Additional File 1: Fitting results of the different modes on the experimental data). In this case, the binding constant (*K_a_*) is equivalent to the quenching constant, which was determined by fitting **Eq. 1 **to the experimental data.(1)

Where *F_0 _*and *F *represent the ﬂuorescence intensities without and with the ligands, respectively; *K_a _*is defined as the binding constant; and [Q] is the concentration of Q. When the concentration of Q is very low, the binding constant *K_a_*, which is equivalent to the equilibrium constant *K*, was calculated at certain experimental temperatures (27 and 37°C). The variation of the binding enthalpy *ΔH*, which was assumed to not change with the temperature, was calculated usingthe classical Van't Hoff equation (**Eq. 2**):(2)

Where *T *is the temperature and *R *the ideal gas constant. The binding free energy *ΔG *was calculated using **Eq. 3**:(3)(4)

The variation of the binding entropy *ΔS *was calculated with **Eq. 4**, and the results are summarized in Table 6[44-46].

**Table 6:** Binding parameters between Q and the three proteins

Pro.	Temp.(°C)	*K_a _*(L/mol)	ΔG (kJ/mol)	ΔH (kJ/mol)	ΔS (J/mol·K)
BSA	27	7.34 × 10^4^	-27.94	5.88	112.80
	37	7.92 × 10^4^	-29.07		
Lys	27	2.93 × 10^4^	-25.65	12.40	126.90
	37	3.44 × 10^4^	-26.92		
Mb	27	3.72 × 10^4^	-26.25	8.08	114.50
	37	4.13 × 10^4^	-27.39		

**Figure 14:**
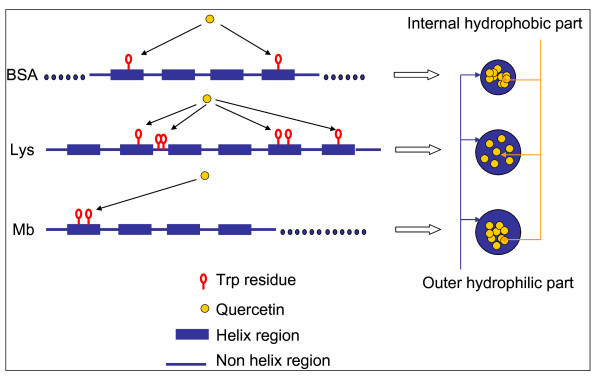
**Schematic thermodynamics of binding Q on different proteins**. Interpretation of the figure is provided in the text.

The negative *ΔG *indicates that the binding of Q and Trp was energetically favourable. The positive *ΔS *and *ΔH *indicates that the binding reactions increased the entropy of the molecular environment of Trp, and were endothermic. This kind of reaction is typically hydrophobic [47]. Six Trp residues are contained in one Lys polypeptide backbone, but only two are contained in BSA or Mb. Although the precise binding location of each Q molecule is yet unknown, the lower entropy values of the BSA and Mb systems indicate that the distribution of Q around Trp residues was more convergent. The higher entropy in the Lys system indicates that the distribution of Q was more scattered, caused perhaps by too many Trp residues. This understanding is illustrated in Figure 14.

### UV-Vis Spectroscopy

**Figure 15:**
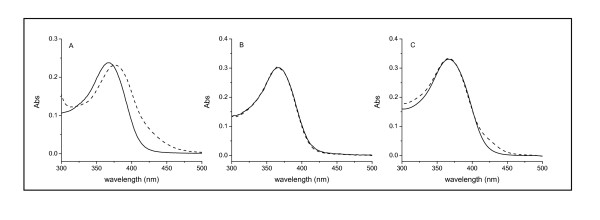
**UV-Vis spectra of free and bound Q to D-BSA, D-Lys, and D-Mb**. The concentration of Q was 1.5 × 10^-5 ^mol/L. The concentration of DMSO was maintained at 10%. The concentration of (A) BSA, (B) Lys, or (C) Mb was 1.5 × 10^-5 ^mol/L. The solid line represents free Q, and the dashed line represents bound Q.

Figure 15 compares the UV-Vis absorption spectra of Q, D-BSA-Q (**A**), D-BSA-Q (**B**), and D-Mb-Q (**C**). The pure Q showed its characteristic band at 367 nm, which is associated with the cinnamoyl group [16]. Normally, the formation of H-bonds between the chromophoric group of Q and auxochromic group can result in an obvious red shift [48-50]; this was found when Q was mixed with BSA (**A**). No shift of this band was found when Q was mixed with Lys (**B**) or Mb (**C**), indicating no H-bonds formed between Q and the two proteins. Thus, the quantity of Q bound to Lys and Mb was probably less than that bound to BSA.

### Binding and Release Capacity of Proteins

**Figure 16:**
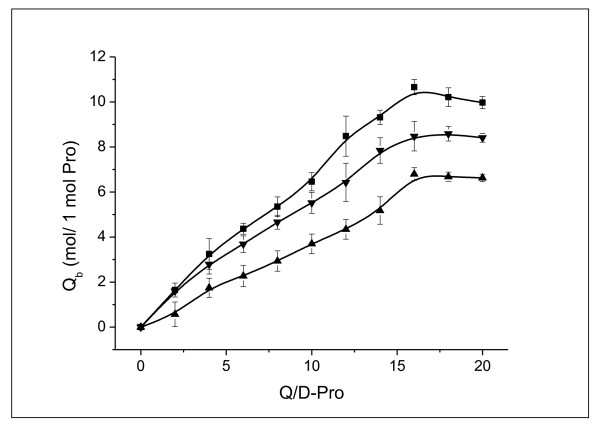
**The Q binding capacities of BSA, Lys, and Mb**. Q_b _represents the quantity of Q bound to protein molecule. The concentration of BSA, Lys, or Mb was all maintained at 1.5 × 10^-5 ^mol/L, and the concentration of DMSO was maintained at 10%. Black square refers to BSA NP; black upper triangle refers to Lys NP; black lower triangle refers to Mb NP.

Figure 16 compares the Q binding capacities of BSA, Lys, and Mb molecules by means of salting-out. The quantities of the bound Q increased with increasing ratio of Q and protein (Q/D-Pro), reaching saturated values (7 for Lys, 9 for Mb, and 11 for BSA) at Q/D-Pro ratios exceeding 16. Thus, one Lys molecule could bind 7 Q molecules, one Mb molecule could bind 9, and one BSA molecule could bind 11. The binding capacity of BSA was confirmed to be the highest. Obviously, H-bonds contributed to the enhanced binding capacity of BSA. In addition, the higher molecular weight (MW) of BSA increased the possibility of surface contact between the protein and Q and favored the hydrophobic effects.

**Figure 17:**
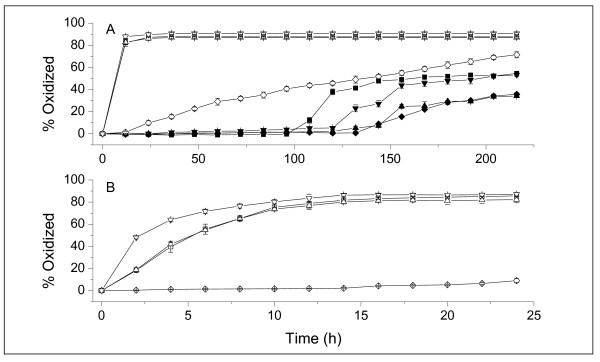
**Comparison of the quantity of the oxidized Q in the system without or with protein**. The concentrations of Q and protein (BSA, Lys, and Mb) were 1.5 × 10^-4 ^and 1.5 × 10^-5 ^mol/L, respectively. Q solution was prepared with 10% DMSO. (A) Measurements during 216 hours. (B) Measurements during the first 24 hours at pH 7.4. Black square refers to Q without protein at pH 1.2; balck rhombus refers to Q with BSA at pH 1.2; black upper triangle refers to Q with Lys at pH 1.2; black lower triangle refers to Q with Mb at pH 1.2; white square refers to Q without protein at pH 7.4; white rhombus refers to Q with BSA at pH 7.4; white upper triangle refers to Q with Lys at pH 7.4; white lower triangle refers to Q with Mb at pH 7.4.

Figure 17 compares the quantity of oxidized Q in the system, without or with proteins, in acidic and neutral conditions (**A**), and shows the enlarged part of the curves at pH 7.4 during the first 24 h of reaction (**B**). Q was rapidly auto-oxidized by O_2 _in water to form o-quinone/quinone methide [13,51-53]. Since only the free Q could be easily oxidized, the curves in Figure 17 are equivalent to the curves of the release capacity of the proteins. Q was relatively stable in acidic conditions, and no oxidation was observed during the first 96 h of the reaction. BSA, Lys, and Mb administration extended the steady state to 120 h. In neutral conditions, Q became very unstable. In Figure 17B, more than 90% of the Q in the system without protein rapidly oxidized during the first 24 h of the reaction. Evidently, the kinetics of oxidation was greatly reduced by the BSA nanocarrier, i.e., less than 10% of the Q was oxidized during the first 24 h of reaction, and less than 70% of the Q was oxidized at 216 h. This protection was not provided by the Lys and Mb nanocarriers.

### Antioxidant Activity of Quercetin

**Figure 18:**
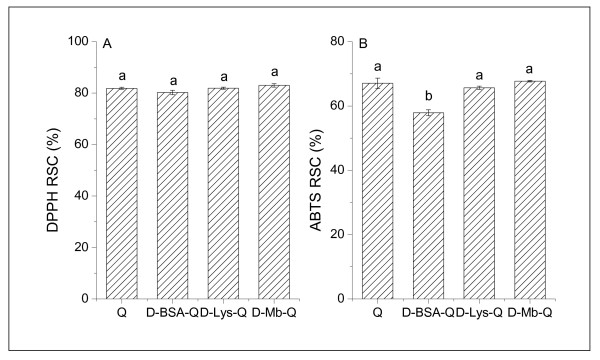
**DPPH and ABTS scavenging activity of Q and embedded Q**. The concentrations of Q was 1.50 × 10^-5 ^mol/L. The concentration of the proteins (BSA, Lys, and Mb) was 1.5 × 10^-6 ^mol/L. The (A) DPPH and (B) ABTS scavenging activities of the proteins were also subtracted from the embedded Q. Markers of different letters in the figure denote that the mean difference is significant at P < 0.05.

DPPH and ABTS radical cation decolourization tests are spectrophotometric methods widely used to assess the antioxidant activity of various substances. Previous studies confirmed that Q has a high DPPH and ABTS antioxidant activity [54-56]. The present study compares the antioxidant activity of Q and embedded Q in BSA, Lys, and Mb nanocarriers. As shown in Figure 18A, the DPPH percent radical scavenging activity (% RSC) of Q was 82%, while the DPPH % RSC of all embedded Q did not change (P < 0.05) at all. Likewise, the ABTS % RSC of Q was 67.06%, while the ABTS % RSC of embedded Q in Lys and Mb nanocarriers did not change (P < 0.05); only the ABTS % RSC of embedded Q in the BSA nanocarriers decreased (P < 0.05) in comparison with free Q. This decrease, however, was so slight that it could be ignored (Figure 18B). Thus, antioxidant activity of Q was not interfered by protein nanoparticles.

Comparing the results acquired from the BSA, Lys, and Mb systems, BSA exhibited the best functional features, such as loading, controlled release, and particularly antioxidant protection of active drugs. Other commercially available flavonoids, such as kaempferol and rutin, were also investigated in order to produce a more general statement and conclusive study of such bionanoparticles. Similar to Q, the thermodynamic, i.e., *ΔG*, values of kaempferol and rutin were negative (both about -30 kJ/mol), and their *ΔH *and *ΔS *were positive (about 6 kJ/mol and 113 J/mol·K for kaempferol, 13 kJ/mol and 130 J/mol·K for rutin, respectively), indicating that these substances could be hydrophobically loaded by BSA since the size of the bionanosystem is less than 30 nm. One BSA could bind 12 kaempferl molecules and 5 rutin molecules. The main features of the oxidation kinetics of kaempferol and rutin in the BSA system were very similar to those of Q under the same conditions.

## Conclusions

In this work, we demonstrated that proteins, such as BSA, Lys, and Mb be used to fabricate bioactive nanoparticles resulting from the secondary and tertiary structure transformations promoted by DMSO to deliver hydrophobic drugs such as Q. The adsorption of Q on proteins was mainly hydrophobic, particularly occurring in the region of Trp residues. BSA exhibited the highest binding capacity of Q, indicating that H-bonding and MWs also contribute to enhancing binding capacity. The formation of a hydrophobic core surrounded by a hydrophilic outer layer was therefore promoted. Protein nanocarriers can not only transport Q molecules, they also provide a protective effect on the activity of Q in both acidic and neutral conditions. The antioxidant activity of Q was also preserved by entrapment by the nanocarrier. Through the formation of complex aggregates composed of proteins, especially the BSA system, DMSO, and Q, such bio-nanoparticles with improved properties could be potentially efficient drug-carriers. Confirmed by further studies on kaempferol and rutin, this approach of protein nanoparticle preparation may provide a general and conclusive way to deliver hydrophobic drugs.

## Methods

### Materials

BSA (Fraction V) (A-0332) was purchased from AMRESCO (Amresco Inc., OH, USA); its MW was 67, 200 Da, and its purity was 98%. Myoglobin (Mb, M0630) was purchased from Sigma Aldrich, Inc. (St. Louis, MO, USA); its MW was 17, 800, and its purity was > 95%. Lysozyme (Lys) was purchased from Sanland Chemical Co. (LTD, LA, USA); its MW was 14, 400 Da. The isoelectric point (pI) of Lys in this work was about 7.0 as determined by zeta potential measurements. The stock solutions of BSA, Lys, and Mb (1.5 × 10^-3 ^mol/L) were prepared with Milli-Q water and stored in the refrigerator at 4°C prior to use. 1-Diphenyl-2-picrylhydrazyl (DPPH, D9132-1G), 2,2'-azinobis (3-ethylbenzothiazoline-6-sulfonic acid) diammonium salt (ABTS, A-1888), and dimethyl sulfoxide (DMSO) were all purchased from Sigma Aldrich, Inc. (St. Louis, MO, USA). The purity of DMSO was 99.5%. Quercetin (3,3',4',5,7-pentahydroxyflavone hydrate, Q-100081) was purchased from the National Institute for the Control of Pharmaceutical and Biological Products (Beijing, China); its purity was 97.3%, as detected by high performance liquid chromatography. The stock solution of Q (1.5 × 10^-3 ^mol/L) was prepared with DMSO, and stored in the refrigerator at 4°C prior to use. All other reagents used were of analytical grade or purer.

### Preparation of DMSO-inducing protein nanoparticle (D-BSA, D-Lys, and D-Mb)

BSA, Lys, and Mb stock solutions (1.5 × 10^-3 ^mol/L) were diluted to 1.5 × 10^-5 ^mol/L; various volumes of DMSO were added. The total volume of the solution was kept at 10 mL, and the concentrations of DMSO were 1%, 10%, 20%, 30%, 40%, 50%, 60%, and 70%. The solution was mixed thoroughly for 5 min. Freeze-drying was used to remove DMSO [57] and obtain the nanoparticles.

### Preparation of Quercetin-loaded protein nanoparticle (D-BSA-Q, D-Lys-Q, and D-Mb-Q)

BSA, Lys, and Mb stock solutions (1.5 × 10^-3 ^mol/L) were diluted to 1.5 × 10^-5 ^mol/L, and various volumes of Q were added. The total volume of the solution was kept at 10 mL, and the concentration of DMSO was kept at 10%; the concentration of Q was adjusted from 1.5 × 10^-5 ^to 1.5 × 10^-4 ^mol/L. The solution was mixed thoroughly for 5 min. Freeze-drying was used to remove DMSO [57] and obtain the nanoparticles.

### Scanning Transmission Electron Microscopy (STEM)

Ten microliter samples were deposited onto a copper TEM grid for 5 s, after which the excess solutions were absorbed. Phosphotungstic acid was used to stain the sample. The observations were performed with a HITACHIS-5500 STEM (Hitachi High-Technologies America, Inc. IL, USA) at 30 KV. Images (1280 × 960 pixels) were acquired using a Gatan high-angle annular bright field (HAABF) scintillating detector.

### Dynamic Light Scattering (DLS) Measurements

Hydrodynamic sizes and zeta potentials were determined by means of photon correlation spectroscopy using a Delsa Nano Particle Analyzer (A53878, Beckman Coulter, Inc., CA, USA). The size measurements were performed at 25°C and at a 15° scattering angle. Size was recorded for 400 μs for each measurement, and the accumulation time was 3 times. In dynamic light scattering, when the hydrodynamic size was measured, the fluctuations in the time of scattered light from particles in Brownian motion were measured. The zeta potential measurements were performed at 25°C. The accumulation time was 70 times, and equilibration time was 60 sec.

### Raman Spectroscopy Measurements

The solution samples were prepared as in the section on sample preparation. Raman spectral data were collected with a HORIBA Jobin Yvon HR800 spectrometer (HORIBA Jobin Yvon S.A.S., Villeneuve Dáscq, France), with 785 nm excitation. Spectral differences were recorded in the 400-2000 cm^-1 ^wave-number range. To increase the signal-to-noise ratio, at least 10 scans of each sample were collected to obtain averaged spectral data. The averaged spectral were baseline-corrected, and smoothed using ORIGIN software (version 8.0). The relative intensities were normalized to the phenylalanine band at 1002 or 1008 cm^-1^.

### Fluorescence Spectrometry Measurements

The fluorescence intensities were recorded with a Cary Eclipse fluorophotometer (Varian, Inc., CA, USA). The widths of the excitation and emission slits of BSA, Lys, and Mb were set to 2.5/5.0, 5.0/5.0, and 10.0/20.0 nm, respectively. All the operations were carried out at 27 and 37°C. Fluorescence spectra were then measured in the range of 200-500 nm at an excitation wavelength of 280 nm. Each spectrum was background-corrected by subtracting the spectrum of the Milli-Q water and DMSO blank.

### UV-Vis Spectrometry Measurements

All the samples were scanned on a Varian Cary 50 UV-visible spectrophotometer (Varian Medical Systems, Inc., CA, USA) at wavelength range of 300-500 nm. The operations were carried out at room temperature, 25°C. The scan rate was 600.00 nm/min. The data interval was 1.00 nm, and the average time was 0.10 sec. All the absorptions of the protein (BSA, Lys, and Mb) were near 280 nm. In the case of Mb, another weak absorption appeared at 420 nm.

### Determination of Quercetin Loading Capacity (Salting Out Analysis)

The Q entrapped by nanocarriers was separated from the free Q through the salting out method as described below. A 5 mL sample was placed in a beaker. Excess ammonium sulphate was added to the beaker, and the mixture was stirred for 10 min and then left to stand for 20 min. A 2 mL solution was transferred to a centrifuge tube, and then centrifuged for 30 min at 15,000 rpm, at 4°C. The absorbance (Abs) of free Q in supernatant was detected at 367 nm by a Varian Cary 50 UV-Vis spectrophotometer (Varian Medical Systems, Inc., CA, USA), and the concentration of free Q was calculated by the standard curve method. The entrapped Q was calculated by determining all the Q in a sample and then subtracting the free Q. All measurements were performed in triplicate.

### Quercetin Stability and Release Study In Vitro (UV-Vis Spectrometry Analysis)

The pH conditions of the release buffer were controlled using phosphate buffer (pH 7.4) or HCl (pH 1.2). The experiment was carried out using an improved method of Arnedo [8] as described below. A 90 mL sample was separated into 30 tubes, placed in an incubator at 37°C, and then wagged at 100 rpm. The tubes were successively detected at predetermined intervals by means of UV-Vis spectrometry. All measurements were performed in triplicate.

### Antioxidant Activity Evaluation

#### DPPH Assay

The DPPH assay was used to evaluate the free radical scavenging activity on the DPPH• of each sample. When DPPH• reacted with an antioxidant compound, the DPPH was reduced. The change in color was measured at 517 nm. The DPPH free radical scavenging activity was determined by the method of Hao [58]. Stock solutions of DPPH were prepared at 2.5 mmol/L, and then diluted to 0.15 mmol/L. Each sample (15 μL) was mixed with 0.05 mol/L (pH 7.4) of Tris-HCl buffer (60 μL) and 0.15 mmol/L DPPH working solution (150 μL) in a 96-well plate. The mixture was shaken vigorously, and then left to stand for 30 min in the dark. The absorbance (*A_Sample_*) at 517 nm was recorded using a microplate reader (Model 680, Bio-Rad Laboratories, Inc., CA, USA). All the samples were analyzed in triplicate. The absorbance of the control (*A_Control_*) was obtained by replacing the sample with ethanol. The percent radical scavenging activity (% RSC) was calculated using the formula shown below:(5)

#### ABTS Assay

The ABTS radical cation decolorization test is a spectrophotometric method widely used for the assessment of antioxidant activity of various substances. The experiment was carried out by the method of Re [59]. In brief, 140 mmol/L ABTS stock solution was diluted in water to a concentration of 14 mM. A mixture of 500 μL 14 mM ABTS diluent and 500 μL 4.9 mM potassium persulfate (KPS) stock solution was placed in a 1.5 mL tube, and then left to stand in the dark at room temperature for at least 12 h before use. To study the samples, the ABTS· solution was diluted with the sample buffer to an absorbance of 0.70 ± 0.02 at 734 nm. After the addition of 900 μL of diluted ABTS· solution to 100 μL of sample, the absorbance (*A_Sample_*) reading was taken after exactly 4 min. A sample buffer blank (*A_Control_*) was run in each assay. All determinations were carried out in triplicate. The percent radical scavenging activity (% RSC) was calculated using **Eq. 5**.

## Competing interests

The authors declare that they have no competing interests.

## Authors' contributions

XJL, HJ, and RF coordinated the experiments, and provided important advice for each. RF performed the majority of the experiments and characterization. ZC, SS, GHZ, FZR, and FL participated in the characterization. All authors read, participated in writing, and approved of the final manuscript.

## Supplementary Material

Additional file 1 **Fitting results of the different modes on the experimental data**. The concentration of BSA (**A **and **B**), Lys (**A' **and **B'**), or Mb (**A'' **and **B''**) were 1.5 × 10^-5 ^mol/L. (**A**), (**A'**), and (**A''**) Comparison of the fitting results of the dynamic, static and simultaneous modes at 27°C. The concentration of Q varied from 0 to 1.2 × 10^-5 ^mol/L. Black square refers to experimental data; dot line refers to the dynamic mode; dash line refers to the static mode; solid line refers to the simultaneous mode. (**B**), (**B'**), and (**B''**) Comparison of the fitting results at 27 and 37°C. Black square refers to 27°C and black round refers to 37°C. Click here for file
