# Supplementary material for: Design and characterization of protein-quercetin bioactive nanoparticles
Source: J Nanobiotechnology. 2011 May 17;9:19. doi: 10.1186/1477-3155-9-19 (PMC3116464; doi:10.1186/1477-3155-9-19)
Supplement: Additional file 1 — Fitting results of the different modes on the experimental data. The concentration of BSA (A and B), Lys (A' and B'), or Mb (A'' and B'') were 1.5 × 10^-5^ mol/L. (A), (A'), and (A'') Comparison of the fitting results of the dynamic, static and simultaneous modes at 27°C. The concentration of Q varied from 0 to 1.2 × 10^-5^ mol/L. Black square refers to experimental data; dot line refers to the dynamic mode; dash line refers to the static mode; solid line refers to the simultaneous mode. (B), (B'), and (B'') Comparison of the fitting results at 27 and 37°C. Black square refers to 27°C and black round refers to 37°C. [file 1477-3155-9-19-S1.DOC]

A’

B’

A’’

B’’

A

B
